# Leveraging environmental microbial indicators in wastewater for data-driven disease diagnostics

**DOI:** 10.3389/fbioe.2024.1508964

**Published:** 2024-11-25

**Authors:** Gayatri Gogoi, Sarangthem Dinamani Singh, Devpratim Koch, Emon Kalyan, Rashmi Rani Boro, Aradhana Devi, Hridoy Jyoti Mahanta, Pankaj Bharali

**Affiliations:** ^1^ Centre for Infectious Diseases, Biological Sciences and Technology Division, CSIR-North East Institute of Science and Technology, Jorhat, India; ^2^ Academy of Scientific and Innovative Research (AcSIR), Ghaziabad, India; ^3^ Materials Sciences and Technology Division, CSIR-North East Institute of Science and Technology, Jorhat, India; ^4^ Advanced Computation and Data Sciences Division, CSIR-North East Institute of Science and Technology, Jorhat, India

**Keywords:** SARS-CoV-2, wastewater-based surveillance (WBS), environmental factors, machine learning (ML), public healh

## Abstract

**Introduction:**

Wastewater-based surveillance (WBS) is an emerging tool for monitoring the spread of infectious diseases, such as SARS-CoV-2, in community settings. Environmental factors, including water quality parameters and seasonal variations, may influence the prevalence of viral particles in wastewater. This study aims to explore the relationships between these factors and the incidence of SARS-CoV-2 across 28 monitoring sites, spanning different seasons and water strata.

**Methods:**

Samples were collected from 28 sites, accounting for seasonal and spatial (surface and intermediate water layers) variations. Key physicochemical parameters, heavy metals, and minerals were measured, and viral presence was detected using RT-qPCR. After data preprocessing, correlation analyses identified 19 relevant environmental parameters. Unsupervised learning algorithms, including K-means and K-medoid clustering, were employed to categorize the data into four distinct clusters, revealing patterns of viral positivity and environmental conditions.

**Results:**

Cluster analysis indicated that seasonal variations and water quality characteristics significantly influenced SARS-CoV-2 positivity rates. The four clusters demonstrated distinct associations between environmental factors and viral prevalence, with certain clusters correlating with higher viral loads in specific seasons. The clustering patterns varied across sample sites, reflecting the diverse environmental conditions and their influence on viral detection.

**Discussion:**

The findings underscore the critical role of environmental factors, such as water quality and seasonality, in shaping the dynamics of SARS-CoV-2 prevalence in wastewater. These insights provide a deeper understanding of the complex interplay between environmental contexts and disease spread. By utilizing WBS and advanced data analysis techniques, this study offers a robust framework for future research aimed at enhancing public health surveillance and interventions.

## 1 Introduction

Wastewater-based surveillance has emerged as a powerful tool for monitoring public health and environmental contamination, playing a pivotal role in the early detection and management of various waterborne diseases and pollutants. This approach, rooted in the analysis of physicochemical parameters within wastewater, has gained importance due to its ability to provide real-time, cost-effective, and community-wide insights into the presence of contaminants. In an era, characterized by the continuous generation of massive amounts of data, harnessing the potential of data-driven approaches has become imperative in wastewater surveillance to enhance its accuracy and efficiency (Wigginton et al., 2015; Mathew and Kanmani, 2020; Srikanth et al., 2019).

The World Health Organization (WHO) emphasizes the importance of effective wastewater management and surveillance in preventing waterborne diseases, such as cholera and typhoid, which continue to pose a significant threat to global public health (World Health Organization, 2019). Traditionally, wastewater surveillance relied on periodic sampling and laboratory testing of water samples, a time-consuming and resource-intensive process. However, recent advancements in sensor technologies and data analysis methods have revolutionized the field by enabling continuous monitoring and analysis of physicochemical parameters in real-time (Newhart et al., 2019).

Physicochemical parameters, including pH, dissolved oxygen, turbidity, and the concentration of specific chemicals, provide critical information about the quality of wastewater. These parameters serve as indicators of potential contamination and can help in the early detection of pollutants, pathogens, and emerging chemical constituents, such as pharmaceuticals and microplastics (Arora et al., 2021).By continuously collecting and analyzing data from various wastewater treatment plants, data-driven approaches can effectively detect abnormal patterns and deviations, thus alerting authorities to potential issues before they escalate into public health crises (Pan et al., 2022; Levin et al., 2024; Moretti et al., 2024). Heavy metal content in wastewater plays a pivotal role in wastewater surveillance by providing essential insights into the overall water quality and potential contamination risks. Monitoring heavy metal concentrations, including elements like mercury (Hg), cadmium (Cd), lead (Pb), selenium (Se), and arsenic (As), contributes to a comprehensive assessment of environmental health and safety of that particular region (Zeiner et al., 2007). These heavy metals are considered priority pollutants due to their toxicity and persistence in aquatic ecosystems (Tchounwou et al., 2012).

The integration of data-driven approaches in wastewater surveillance leverages the power of machine learning, artificial intelligence, and big data analytics. These technologies enable the development of predictive models that can forecast contamination events, optimize treatment processes, and guide effective policy decisions. Such models learn from historical data, adapt to changing conditions, and provide actionable insights that empower decision-makers to respond proactively to emerging challenges in wastewater management (Moretti et al., 2024; Sahu et al., 2023; Van der Werf et al., 2023). High mutation rates lead to analytical limitations, requiring frequent updates to primers and probes used in RT-PCR assays. Moreover, wastewater samples contain complex microbial communities that may hinder variant identification accuracy. Other challenges include the need for improved data resolution to differentiate among closely related variants, which is vital for effective public health responses and anticipating variant-driven case surges (Thakur et al., 2022; Gogoi et al., 2024).

Machine learning (ML) and Deep Learning (DL) models have been employed for time-series predictions and track COVID-19 outbreaks in multiple communities as well as pre-screening tool for the identification of differences among the variant composition of different wastewater samples (Ai et al., 2022; Férez et al., 2023). Utilizing unsupervised ML algorithms, a quantifiable model for characterizing peaks and gaps in multiple waves of COVID-19 across 120 countries not only reveals the complexity in predicting growth or decline rates within each wave, but also identifying common features among the clusters, offering potential insights into anticipating future developments (Mahanta and Narahari Sastry, 2022).

Extensive seroepidemiological and genomic investigations for SARS-CoV-2 have been conducted across India, encompassing smaller regions like Jorhat district of Assam in the north east India. These studies provided evidence of a significant number of positive cases in the area that are linked to common Omicron and Delta variations (Naushin et al., 2021; Wahengbam et al., 2023). These studies have delved into the dynamics of COVID-19 progression within the Indian population, employing transcriptomic data analysis. Further studies also reveal serological surveys in the north eastern region of India which involves ML approach to discern infection statuses among Covaxin recipients (Kshattry et al., 2022; Singh et al., 2022). These studies show the potential of data-driven ML approaches for deciphering complex questions in diverse epidemiological studies.

This study aims to investigate how data-driven approaches can augment wastewater surveillance by correlating physicochemical parameters and heavy metals content with incidences of viral loads. This correlation study will indicate the early detection of the new pandemic for a particular region. It seeks to improve the early detection of contaminants and abnormal patterns, thereby strengthening public health and environmental safety.

## 2 Materials and methods

### 2.1 Wastewater sample collection

**FIGURE 1:**
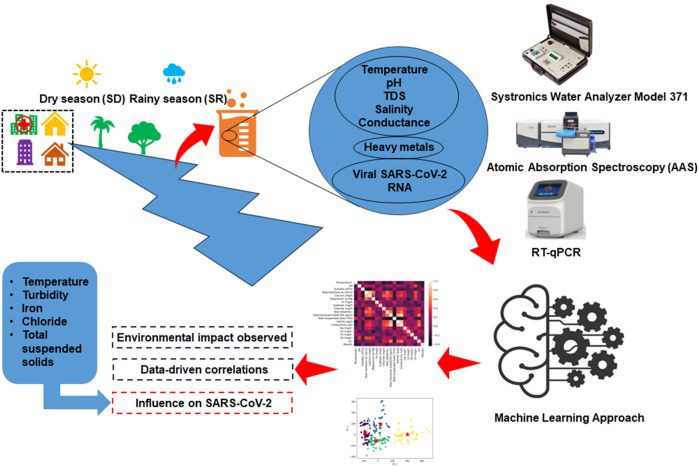
Overview of the study methodology, detailing the process from sample collection, through various stages of data processing, to final outcome representation. The diagram highlights key steps, including environmental sampling, laboratory analysis, data preprocessing, and clustering analysis for interpreting the results.

The North-East region of India is a captivating region consisting of eight states, each brimming with unique cultural and geographical richness and shares international borders with Bhutan, China, Myanmar, and Bangladesh. Assam is the second largest state out of these eight states and share borders with all others. The current study was conducted in Jorhat district of Assam which has diverse indigenous communities, residing within low-resource and low-income settings. These communities heavily rely on medical facilities situated within their respective localities. These facilities serve as lifelines, providing essential healthcare support to those people. Wastewater samples have been collected from 28 different sites stretched across Jorhat district, representing three settings: healthcare, residential, and river bodies. Sampling was carried out during both rainy and dry seasons, spanning from September 2022 to March 2023. Two water layers were considered for sampling, the surface layer and the intermediate layer (30 cm depth) which resulted into four spatiotemporal conditions: surface layer during the rainy season (SR), intermediate layer during the rainy season (IR), surface layer during the dry season (SD), and intermediate layer during the dry season (ID). The samples were manually collected in Polypropylene bottles (PP). Sampling took place during morning hours to capture the viral peak load, and no rainfall was reported within the 24 h prior to collection. Additionally, we measured various physicochemical parameters, including temperature, pH, total dissolved solids (TDS), salinity, and conductance, using a Systronics Water Analyzer Model 371 during the sampling process (Férez et al., 2023; Yadav and Chauhan, 2023) and the analysis of heavy metals was carried out by using Atomic Absorption Spectroscopy (AAS) manufactured by Analytikjena model Zeenit 700p. Sample preparation involves careful collection, labelling, and homogenization of environmental samples, with subsequent digestion using appropriate acids. Certified reference standards are employed to create a calibration curve, establishing the relationship between absorbance and known heavy metal concentrations. Instrument setup encompasses optimizing AAS parameters, including lamp current, wavelength, and slit width, while regular alignment checks ensure instrument accuracy and standard limit of quantification for the selected element is upto 1,000 mg/L. In the measurement procedure, the sample is aspirated into the AAS instrument, and the absorbance is recorded at the specific wavelength for each heavy metal of interest. We have selected mercury (Hg), lead (Pb), cadmium (Cd), selenium (Se), and arsenic (As) for the present study (Zeiner et al., 2007). The complete methodology, from sample collection through processing to outcome representation, is illustrated in Figure 1.

### 2.2 Wastewater sample processing

The wastewater samples were processed in a BSL2 environment with strict adherence to personal protective equipment (PPE) protocols to ensure the workers safety (Spurbeck et al., 2021). The sample processing steps consisted of Sample Homogenization and Pasteurization, Sample Filtration, and Sample Concentration using PEG-NaCl Method followed by viral extraction (Supplementary Figure S1). These meticulous steps ensured the proper processing and concentration of wastewater samples, while stringent safety measures were in place throughout the procedure (Smyth et al., 2022).

### 2.3 Viral RNA extraction and reverse transcription polymerase quantitative polymerase chain reaction (RT-qPCR)

The viral RNA extraction from wastewater samples was carried out using the QIAamp^®^ Viral RNA Mini Kit (250) from Qiagen, Germany. The extraction process adhered strictly to the manufacturers’ instructions, with a focus on obtaining SARS-CoV-2 viral nucleic acid from the PEG pellets post-virus concentration. In the 40 mL protocol, the RNA was eluted to a final volume of 40 µL and stored at −20°C when immediate processing was not possible, although every effort was made to process the samples on the same day.

Subsequently, the extracted RNA samples underwent analysis through reverse transcription-quantitative polymerase chain reaction (RT-qPCR) conducted on the QuantStudio™ 5 real-time PCR system by Applied Biosystems™ Inc., United States. The RT-qPCR assay targeted specific genes, namely, ORF-1ab and N genes, in a confirmatory test using the CoviPath™ COVID-19 RT-qPCR Kit from Applied Biosystems™ Inc., United States. Each 25 µL PCR reaction mixture consisted of 10 µL of the RNA extract, 6.25 µL of CoviPath™ COVID-19 Assay Multiplex, 1.25 µL CoviPath™ 1 Step Multiplex Master Mix (No ROX™), and the volume was adjusted to 25 µL using molecular grade water supplied by Sisco Research Laboratories Pvt. Ltd. Negative controls utilized ultrapure nuclease-free water, and for positive controls, CoviPath™ COVID-19 was employed, following the manufacturer’s dilution guidelines.

The RT-qPCR reactions were carried out with an initial step at 53°C for 10 min, followed by 95°C for 2 min, and then cycled 40 times at 95°C for 3 s and 60°C for 30 s in the QuantStudio™ 5 real-time PCR system. Notably, the interpretation of SARS-CoV-2 positivity in this study underwent a revision, aligning with the CoviPath protocol. A cycle threshold (Ct) value of 35 was adopted as the criterion for positivity, ensuring a standardized and reliable approach for the detection and characterization of SARS-CoV-2 variants. This modification facilitates downstream Whole Genome Sequencing (WGS) analysis, making the study a more consistent and robust method for SARS-CoV-2 variant identification (Spurbeck et al., 2021; Ravi et al., 2024).

### 2.4 Quantitative analysis through machine learning

#### 2.4.1 Correlation analysis of features

The water samples analysis and RT-qPCR tests generated 27 parameters out of which 24 parameters were distinct features as independent parameters and 3 features as dependent variables (ORF 1 ab, N gene, and RNaseP) in building the dataset for training the ML models. During initial preprocessing it was found that 5 out of the 24 features suffered from missing value and noisy value problem. Due to the complexity of the problem as well as size of data, imputation methods were refrained from being used and all these 5 features were not considered further. The remaining 19 features were then subjected to find their correlation with the three dependent variables and the final RT-qPCR outcome using Sparman’s rank correlation analysis. This correlation will help to find the monotonic relationship between each feature with the dependent variables as well as with one another. This is a prominent approach of feature engineering which helps to identify the most relevant features for modelling. The value ranges from −1 to 1, where −1 and 1 resembles a perfectly negative and perfectly positive correlation respectively with 0 indicating no linear correlation.

#### 2.4.2 Partitional clustering of sample sites

Clustering is a fundamental unsupervised machine learning technique used to discover hidden structures or patterns within a dataset. It involves grouping similar data points into clusters, where the members within a cluster are more similar to each other than to those outside the clusters. This is an iterative process which continues to form a new set of clusters till the optimum result is obtained. In the current study, K-means has been employed for interpretation of the underlying data. The K-means clustering method groups data points into clusters based on their similarity. It partitions data into K clusters, aiming to minimize within-cluster sum of squares. The algorithm starts with random cluster centroids and iteratively assigns data points to the nearest centroid, then updates the centroids based on the assigned points, repeating until convergence. During the assignment, each point is assigned to the nearest cluster mean with the least Euclidean distance such that,

$$S_{i}^{\left(t\right)}=\left\{x_{p}:{\left\|x_{p}‐m_{i}^{\left(t\right)}\right\|}^{2}\leq {\left\|x_{p}‐m_{j}^{\left(t\right)}\right\|}^{2}\forall_{j},1\leq j\leq k\right\}$$ (1)

Where the point *xp* is assigned to exactly one *S(t)*.

The centroids are then recalculated and the observations are reassigned to new clusters. This process is repeated till the algorithm converges and reaches an optimal state.

$$m_{i}^{\left(t\right)}=\frac{1}{\left|S_{i}^{\left(t\right)}\right|}\sum_{x_{j}\in S_{i}^{\left(t\right)}}x_{j}$$ (2)

K-means is widely used for data segmentation, pattern recognition, and feature engineering in various fields (Ahmed et al., 2021; Pearson, 1895).

#### 2.4.3 Elbow method for optimal number of clusters

The Elbow Method is a widely used heuristic method for determining the optimal number of clusters (K) in a K-means clustering analysis. It involves performing the K-means clustering algorithm on the dataset for a range of K values and evaluating within-cluster sum of squares (WCSS) for each K. The WCSS is a measure of the total variance within the clusters. The point at which the reduction in WCSS starts to slow down, forming an “elbow” in the plot, is considered the optimal K value (Arthur and Vassilvitskii, 2007). WCSS is computed by,

$$WCSS=\sum_{i=1}^{K}\sum_{x\in C_{i}}{\left\|x-c_{i}\right\|}^{2}$$ (3)

Where K is the number of clusters, C_i_ represent the *i^th^* with *ci* as the centroid of the *i^th^* cluster and *x* is any data point. As the number of clusters increases, the WCSS will generally decrease, as each data point will be closer to its cluster centroid. However, at a certain point, the WCSS will start to plateau, as increasing the number of clusters will no longer significantly reduce the distance between data points and their cluster centroids. The elbow in the WCSS plot indicates this point, and the optimal number of clusters is the value of K at the elbow (Rdusseeun and Kaufman, 1987).

#### 2.4.4 Performance metrics

A number of cluster validation metrics have been used in this study to evaluate the performance and quality of the clusters generated by the two clustering algorithms, K-means and K-medoid clustering.

##### 2.4.4.1 Silhouette score

This is the measure of how similar an object is to its own cluster (cohesion) compared to other clusters (separation). Its value ranges from −1 to 1, where higher values indicate better-defined clusters.

$$S\left(i\right)=\frac{\left(b\left(i\right)-a\left(i\right)\right)}{\max\left(b\left(i\right),a\left(i\right)\right)}$$ (4)

Where, *S(i)* is the silhouette score for data point *i*, *a(i)* is the average distance between *i^th^* data point and other data points in the same cluster, and *b(i)* is the minimum average distance, minimised across clusters, between *ith* data point and the data points in a different cluster.

##### 2.4.4.2 Davies-Bouldin index (DBI)

DBI estimates the average similarity between each cluster and the most comparable one. Lower numbers imply better grouping.

$$DBI\left(C\right)=\frac{1}{K}\sum_{i=1}^{K}\max_{i\neq j}\frac{∆\left(C_{i}\right)+∆\left(C_{j}\right)}{\delta C_{i},C_{j}}$$ (5)

Where the intra-cluster distance is represented by ∆(*Ci*) and the inter-cluster distance by δ (*Ci*, *Cj*).

##### 2.4.4.3 Dunn index (DI)

DI is the measurement of the compactness of clusters and the separation between them. Higher values are better, indicating better-defined clusters.

$$DI=\frac{\min\left(\delta \left(Ci,Cj\right)\right)}{\max\left(\Delta \left(Ci\right)\right)}$$ (6)

Where, δ (*Ci, Cj*) represents the minimum intra-cluster distance between clusters *Ci* and *Cj*, while Δ(*Ci*) is the diameter of cluster *Ci*. Better clustering is indicated by a higher DI, which denotes tightly packed, well-separated clusters.

##### 2.4.4.4 Inertia

Inertia measures the overall compactness of clusters through total squared distance between each data point and the cluster centre. Tighter and better-defined clusters are indicated by lower inertia levels and *vice versa*. Inertia is one of the key ideas in evaluating the quality of clustering solutions which is frequently used in conjunction with techniques like the Elbow Method to establish the ideal number of clusters (Hastie et al., 2009; Xu and Tian, 2015).

$$inertia=\mathrm{argmin}\sum_{i=1}^{k}\sum_{x\in S_{i}}{\left\|x-\mu_{i}\right\|}^{2}$$ (7)

Where *S* is a set of observations with *x* as a data point and *μ* as the mean. Any distance metric, including the cosine, Manhattan, and Euclidean distances, may be used to compute the inter-cluster distance. Usually, the greatest distance between any two locations in the cluster is used to compute the intra-cluster distance.

These metrics assess the compactness, separation, and general cohesiveness of the clustering findings and allow to statistically assess the suitability and efficacy of the clustering techniques while making it easier to choose the best algorithm for the particular dataset. As the ground truth was not known, these metrics would help in converging that the clusters formed were correctly formed.

## 3 Results

### 3.1 Sample collection and analysis

For this study, 448 wastewater samples were collected from 28 different sites within Jorhat district of Assam, India (Supplementary Figure S2) during different season (Supplementary Table S1). Among these samples, 144 were reported to be positive for SARS-CoV-2 with an illustrative portrayal of the distribution of SARS-CoV-2 ORF 1ab, N, and RNAseP genes, alongside the corresponding results indicating positivity and negativity in wastewater samples (Supplementary Figure S3). The analysis covered a range of physicochemical parameters, including temperature, pH, turbidity (NTU), total hardness as CaCO_3_, calcium (mg/L), magnesium as Mg, iron (Fe) content (mg/L), sulphate (mg/L), chloride (mg/L), total alkalinity, total dissolved solids (TDS) in ppm, total suspended solids (TSS), salinity (ppt), and conductivity (µS). Additionally, the study examined the presence of heavy metals such as Hg (mercury), Cd (cadmium), Pb (lead), Se (selenium), and As (arsenic) in the samples, providing a comprehensive overview of the environmental conditions and the presence of SARS-CoV-2 in the region.

### 3.2 Correlation analysis

**FIGURE 2:**
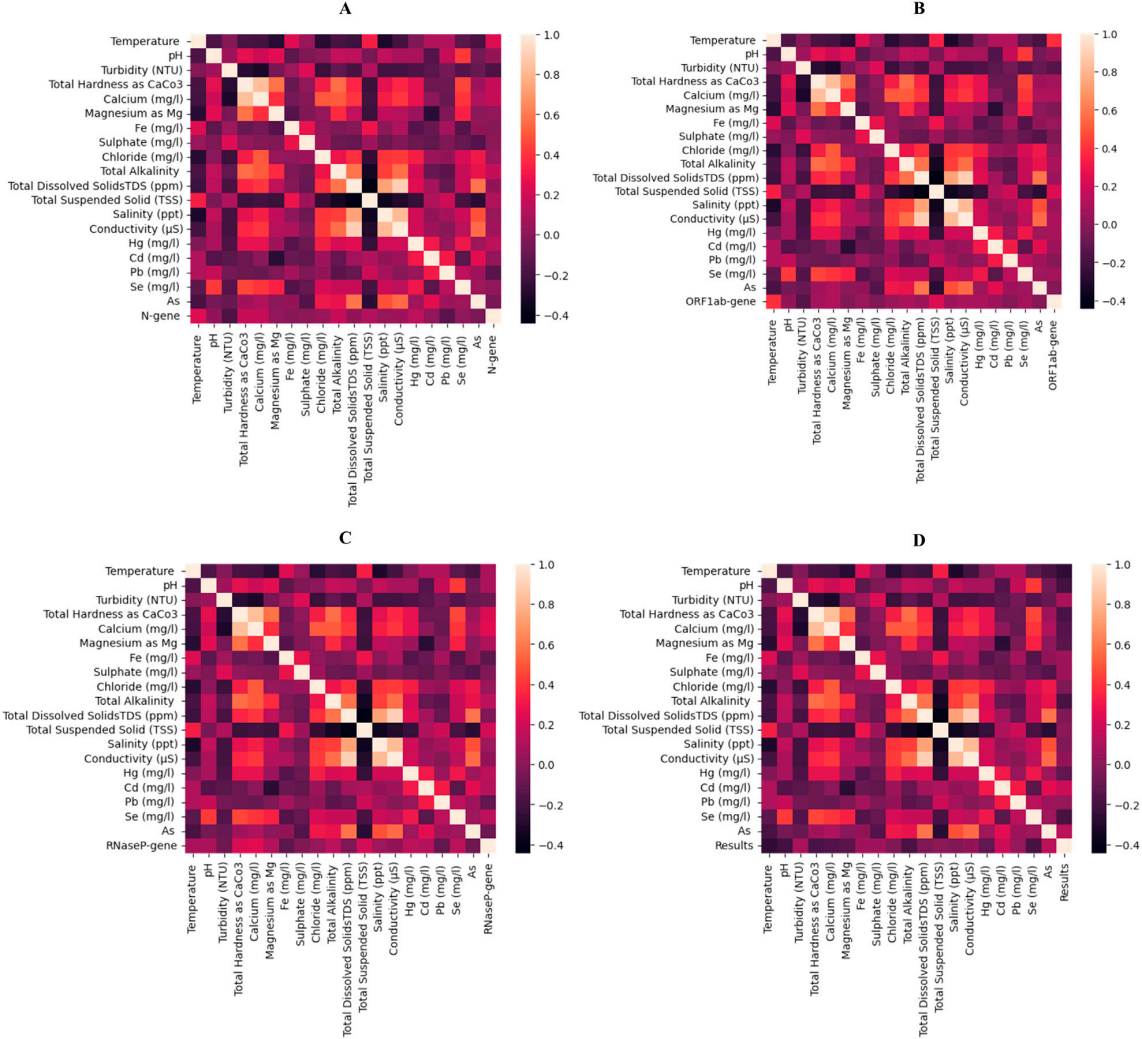
Heatmap represented Spearman’s rank correlation of all the physicochemical properties considered as independent features in this study. The correlation plots are depicted for **(A)** N gene, **(B)** ORF1ab gene, **(C)** RNaseP gene and **(D)** for RT-qPCR results. The plot showing the presence of both + ve and -ve correlation with the selected features.

The independent features considered in this study were subjected to Spearman’s rank correlation with the dependent parameters, i.e., N gene, ORF 1 ab, RNaseP and the RT-qPCR result (positive and negative). In correlation with N gene, features such total hardness, Magnesium content and total alkalinity had high positive correlation (≈0.22) compared to temperature, pH, calcium, and Cadmium (≈0.15). Whereas, turbidity, Iron and lead showed a negative correlation (≈– 0.14) with N gene. For the ORF 1ab gene, temperature displayed higher positive correlation (≈– 0.27) compared to other features like pH, total hardness, Calcium, Magnesium, total alkalinity, TSS and Selenium (≈– 0.14). Whereas, Arsenic had a negative correlation of ≈ – 0.17. Similarly, in case of RNaseP gene it was seen that total hardness and calcium had high positive correlations (≈0.27 and ≈0.25 respectively) as compared to other features like pH, Magnesium, alkalinity and TSS. Whereas, features like Iron, Lead and Arsenic contents had a negative correlation (≈– 0.12). Considering the above correlation analysis, it can be seen that most of the features are correlated with the dependent features and will provide insights and contribute in dividing sample locations into relevant clusters. Figure 2 depicts all these findings in the form of heat maps with each dependent features as well as the final outcome, i.e., positive and negative results of RT-qPCR.

### 3.3 Clustering of sample sites with k-means

The elbow curve (Equation 3) which plots the variance or WCSS against the number of clusters is used to determine the optimal cluster size for a given dataset. The idea is to find the “elbow” point in the curve, which represents the point where increasing the number of clusters ceases to significantly reduce the variance. In the current study, the choice of four clusters was driven by the point on the curve where further increasing the number of clusters resulted in diminishing returns in terms of reducing variance (Supplementary Figure S4) making them adequate to capture the underlying patterns in the present dataset.

**FIGURE 3:**
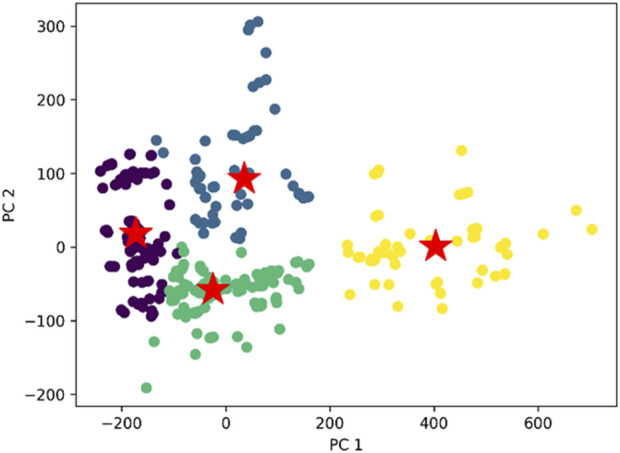
The comprehensive plot of K- means clustering with distinct four clusters and their centroids. The cluster we obtained with respect to the two principal component PC1 and PC2 derived using principal component analysis (PCA).

K-means clustering (Equations 1, 2) was then to the dataset of this study which resulted in the formation of four distinct clusters (Figure 3). Within this visualization, four unique clusters are delineated by different colours, each serving as a visual indicator of the distinct groupings within our dataset. To signify the centroid, or the central point, of each cluster, we have marked it with a red and green star (Rdusseeun and Kaufman, 1987; Hastie et al., 2009).

### 3.4 Performance evaluation

The performance of the clustering algorithms used in this study were measured with four metrics Silhouette coefficient, Davies-Bouldin indicator, Inertia, and Dunn Indicator (Equations 4–7). These metrics serve as valuable tools to evaluate the cohesion, separation, and overall effectiveness of the clusters generated by each algorithm, aiding us in making informed and data-driven conclusions about the quality of our clustering solutions.

**FIGURE 4:**
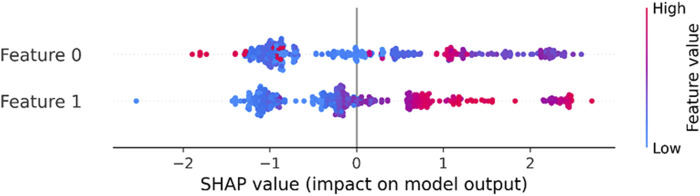
SHAP dependence plot of the features (in terms of PC1 and PC2) of the k-means clustering model.

The Silhouette coefficient, which assesses cluster quality, produced values of 0.38 for K-means indicating comparable cluster cohesion and separation (Table 1). Likewise, the Davies-Bouldin indicator, a measure of cluster compactness and separation, returned values of 0.78 demonstrating closely aligned results. In our study, we have chosen to focus on samples that overlap in both algorithms, specifically selecting those data points for further analysis. The contributions of the principal components in the model’s prediction and identifying the patterns in the dataset has been depicted through SHAP dependence plot in Figure 4.

**TABLE 1:** Performance analysis of clustering algorithms. These analyses were done to verify if the clusters were formed correctly. Silhouette coefficient, Davies-Bouldin indicator.

Silhouette coefficient	Davies-bouldin indicator	Inertia	Dunn indicator
0.38	0.78	3.3 × 10^6^	1.30

### 3.5 Analysis of the clusters

With k-means clustering, the 448 samples were categorized into four distinct clusters: cluster 0, cluster 1, cluster 2, and cluster 3. Notably, our analysis revealed that the highest rate of viral positivity was observed within cluster 2, whereas the lowest rate was found in cluster 0 (Table 2). These findings offer valuable insights into the distribution of SARS-CoV-2 within our sample population, shedding light on potential patterns or associations that may be of significance in the context of the study’s objectives.

**TABLE 2:** Cluster details with viral positivity from K- means algorithms.

Cluster	Total samples	Positive samples	Rainy (%)	Dry (%)	Surface (%)	Intermediate (%)
Cluster 0	160	42 (26.25%)	9 (21.43%)	33 (78.57%)	16 (38.09%)	26 (61.90%)
Cluster 1	63	19 (30.16%)	3 (15.79%)	16 (84.21%)	11 (57.89%)	8 (42.11%)
Cluster 2	70	33 (47.14%)	28 (84.84%)	5 (15.15%)	14 (42.86%)	19 (57.14%)
Cluster 3	136	43 (31.62%)	31 (72.09%)	12 (27.91%)	23 (53.49%)	20 (46.51%)

*Rainy, and dry are two different season and two water layers, the surface and the intermediate layer (30 cm depth).

Cluster 2, which exhibited the highest SARS-CoV-2 positivity rate (47.14%), with a remarkable 84.84% during the rainy season. Interestingly, we also noted that the intermediate layer of water displayed a substantial positivity rate of 57.57%, whereas the surface layer showed a slightly lower rate of 42.42%. Furthermore, this cluster consisted of the samples which were collected from sources at comparatively the higher average temperature (24.98°C) than other clusters. Notably, we observed that turbidity, sulphate, total alkalinity, and total dissolved solid (TDS) content were relatively lower in this cluster when compared to the other clusters. Conversely, factors such as iron, chloride, total suspended solid (TSS), and conductivity were found to be higher in this cluster. The combination of environmental factors within this cluster could potentially contribute to the elevated viral positivity observed, which may show relationship between environmental conditions and leading to the SARS-CoV-2 prevalence in this cluster in our study (Férez et al., 2023; Xu and Tian, 2015; Bishop, 2006; Kisand et al., 2023; Vasickova et al., 2010; Osborne et al., 2022; Weller, 2020).

In contrast, cluster 0, which exhibited the lowest positivity rate (26.25%), a higher positivity rate of 78.57% during the dry season which contrasts with cluster 2. Both cluster 2 and cluster 0 displayed a similar pattern where the intermediate water layer had a higher positivity rate at 61.90%, compared to 38.09% in the surface layer. Cluster 0 notably had the highest turbidity content at 17.69 NTU among all clusters, and it also showed higher levels of magnesium, sulphate, total alkalinity, and TDS content in comparison to the other clusters. However, iron, chloride, and TSS were comparatively lower in cluster 0. This distinctive combination of water quality parameters may contribute to the observed lower positivity rate within this particular cluster (Férez et al., 2023; Bishop, 2006; Kisand et al., 2023; Vasickova et al., 2010; Osborne et al., 2022; Weller, 2020).

In the remaining two clusters, i.e., cluster 1 and cluster 3, the SARS-CoV-2 positivity rates exhibited a relatively consistent range, with values falling between 30.15% and 31.61%, respectively. Notably, both clusters shared similar environmental conditions, with samples temperatures ranging from 22.55°C to 23.77°C and pH levels hovering between 7.09 and 6.97. Moreover, key water quality parameters such as total hardness as CaCO_3_, magnesium, iron, chloride, total alkalinity, TDS, salinity, and conductivity demonstrated comparable values in both clusters. However, a significant distinction emerged in the levels of sulphate and TSS, with cluster 3 exhibiting higher concentrations of these elements in the water when compared to cluster 1. These nuanced differences in water quality factors may contribute to the slight variations observed in SARS-CoV-2 positivity rates between cluster 1 and cluster 3. (Férez et al., 2023; Bishop, 2006; Kisand et al., 2023; Vasickova et al., 2010; Osborne et al., 2022; Weller, 2020).

While analysing the physicochemical properties among the clusters, it was seen that there were distinct variations across each cluster (Supplementary Figures S5–S7). This observation may suggest that these specific parameters could potentially exert an influence on the pattern of SARS CoV-2 positivity. The diverse clusters exhibited in the figures underscore the significance of these parameters in understanding and potentially predicting the occurrence and spread of the virus.

### 3.6 Introspection of overlapping sample sites

In our analysis, we have observed that a few sample sites were associated with multiple clusters, such as 0/3, 1/3, 0/2, 1/2, 2/3, 0/1/2, and 0/2/3 for different samples of same site. After minute examination, it was found that the combination of clusters 0 and 3 appeared most frequently (8 different sites) making it the most prevalent two-cluster combination. Additionally, the combination of clusters 0, 1, and 2 occurred 4 times, which is the most common three-cluster combination in our observations (Table 3).

**TABLE 3:** Details of the sites and number of samples comprising 0/1/2 cluster combination.

Locations	Cluster 0		Cluster 1		Cluster 2	
	Rainy	Dry	Rainy	Dry	Rainy	Dry
Athuvoga bridge (H S)	0	2	0	6	8	0
JMCH Hospital Outlet (H S)	1	1	4	4	3	3
Tarajan Kakoty gaon (H S)	3	0	0	8	5	0
Teok Tea Estate (H S)	1	8	1	0	6	0

*HS, hospital site.

Further introspection of sites in the 0/3 cluster combination revealed that out of the 8 sites, 6 of them (75%) are situated near flowing water sources such as rivers and streams, while the remaining 2 (25%) are near stagnant water sources (Table 4). Similarly, for the sites associated with the 0/1/2 cluster combination, it was found that all 4 of them (100%) are in close proximity to medical colleges and hospitals (Supplementary Table S2). On a positive note, this shows that the clusters formed out of the sample sites also had similar patterns of data on similar geographical positionings.

**TABLE 4:** Details of the sites and number of samples comprising 0/e cluster combination.

Locations	Cluster 0		Cluster 3	
	Rainy	Dry	Rainy	Dry
Kuhum stream (Rv S)	4	6	4	2
Kamarbandha (Rv S)	4	8	4	0
CID, CSIR-NEIST (R S)	0	6	8	2
Bhogdoi river (Rv S)	0	8	8	0
FRU, Teok (R S)	2	8	6	0
Baghmora (Rv S)	5	8	3	0
Jhanji, Jorhat-Sibsagar border (Rv S)	1	8	7	0
Nimatighat (Rv S)	0	8	8	0

* RvS, river site; RS, residential site.

**FIGURE 5:**
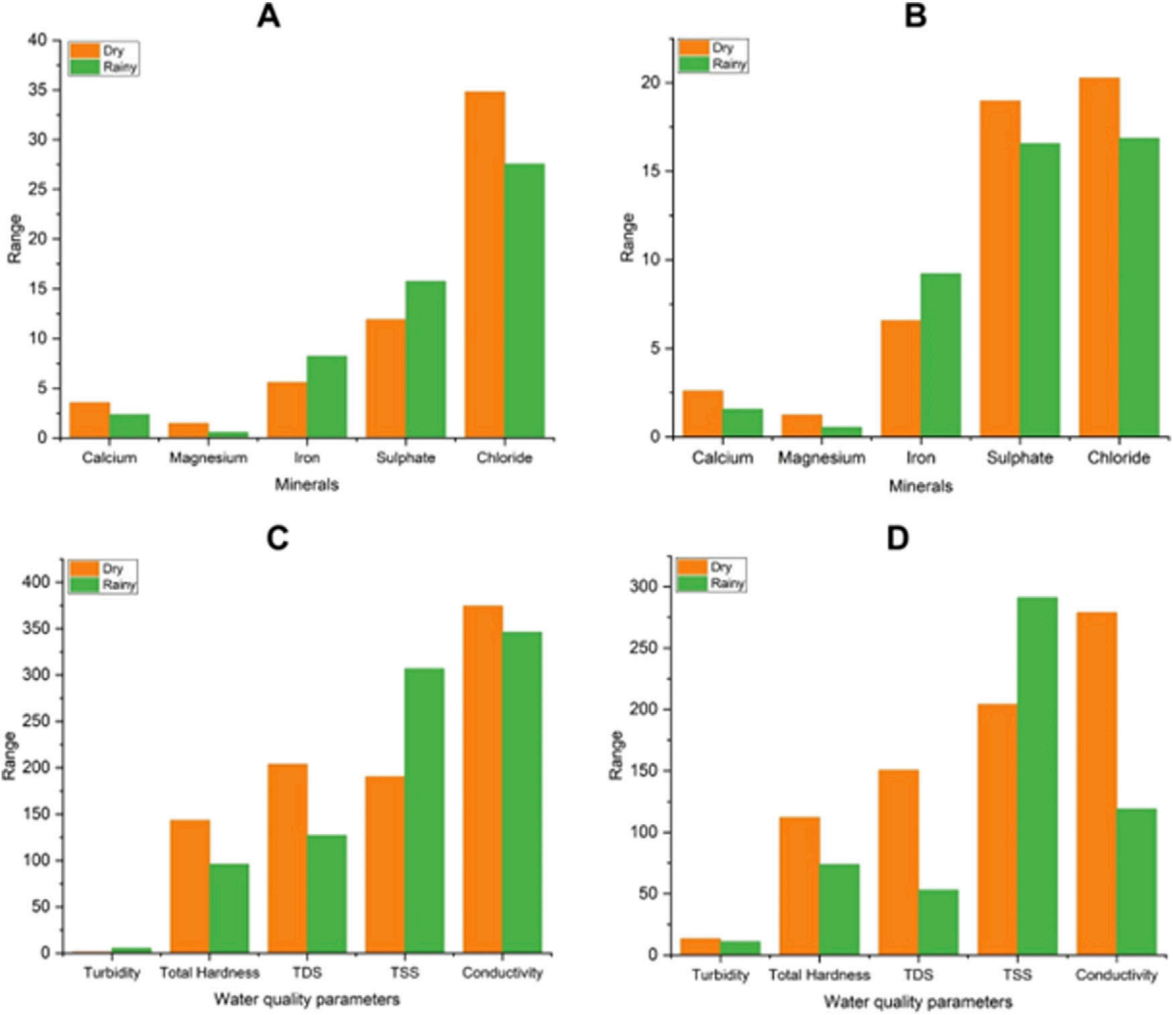
Variation in minerals and water quality parameters with season for changes in clusters. **(A)** variation in mineral content of sites for cluster change from 0/1 to 2 **(B)** variation in mineral content of sites for cluster change from 0/1 to 3 **(C)** variation in water quality parameters of sites for cluster change from 0/1 to 2 and **(D)** variation in water quality parameters of sites for cluster change from 0/1 to 3.

Further, we have observed that some of the sample sites (17 out of 28) were associated with multiple clusters, such as 0/3, 1/3, 0/2, and 1/2 with seasonal variation. The sites which were in cluster 0 and 1 during dry season shifted to cluster 2 and 3 during the rainy season. In this regard it was seen that the physicochemical parameters of the sites showed variations with the season. The sites which changed the cluster from 1 to 2 during dry to rainy season had decline values in total hardness, TDS, conductivity, Calcium, Magnesium whereas elevated values in turbidity, TSS, Iron and Sulphate. Similarly for sites which changed cluster from 0 to 2 had decline in values of hardness, TDS, calcium, magnesium and elevated values in iron, chloride, and TSS. For the sites which changes the clusters from 0 or 1 to 3 during dry to rainy season had decline values in most of the parameters like total hardness, TDS, conductivity, calcium, magnesium, sulphate, chloride but elevated values in iron and TSS (Figure 5).

## 4 Discussion

The present study utilized unsupervised machine learning algorithms to analyse a dataset comprising physicochemical parameters, heavy metal content, and SARS-CoV-2 positivity data from wastewater samples that has collected from 28 different sites in Jorhat district, Assam, India. To our present understanding, this study stands as a pioneering endeavour within the North Eastern region of India, marking the inaugural exploration into the influence of environmental variables on SARS-CoV-2 positivity through the application of a machine learning framework. This methodological approach represents an innovative step in comprehending the intricate interplay between environmental dynamics and viral prevalence, offering novel insights in this unexplored geographical domain (Cabral, 2010; Rimoldi et al., 2020).

The clustering results indicated K-means algorithm has effectively partitioned the data into four clusters, demonstrating comparable cluster quality as assessed by the Silhouette coefficient and Davies-Bouldin indicator. The clusters’ differential characteristics elucidated potential associations between environmental conditions and viral prevalence. Notably, Cluster 2 displayed the highest SARS-CoV-2 positivity rates, especially during the rainy season and in the intermediate water layer. This cluster demonstrated higher temperatures, lower turbidity, sulphate, alkalinity, and total dissolved solids but higher concentrations of iron, chloride, and total suspended solids, hinting at specific environmental conditions favouring increased viral presence. These observations resonate with prior research highlighting the impact of temperature and water quality on viral persistence (Mahanta and Narahari Sastry, 2022; Rimoldi et al., 2020; Ahmed et al., 2020). Conversely, Cluster 0 exhibited the lowest positivity rates and distinct water quality parameters, notably higher turbidity and concentrations of certain minerals and ions. The distinctiveness of these clusters underscores the potential influence of environmental factors in shaping viral prevalence patterns within wastewater.

We further focused our analysis on samples that overlapped in during clustering, which resulted in the selection of 429 data points for further study. Among these samples, 31.93% tested positive for SARS-CoV-2, while the remaining 68.06% tested negative. Notably, our analysis revealed varying rates of viral positivity across the four clusters, with Cluster 2 exhibiting the highest rate of 47.14% during the rainy season, and Cluster 0 displaying the lowest rate of 26.25%, particularly during the dry season. Environmental factors, such as temperature, turbidity, water layer, and specific water quality parameters, were found to vary across the clusters, potentially influencing the observed SARS-CoV-2 positivity rates. Our findings suggest a potential link between environmental conditions and the prevalence of SARS-CoV-2 in different clusters. Additionally, we observed that certain sample sites were consistently associated with specific cluster combinations, indicating potential spatial patterns and associations. For instance, sample sites near flowing water sources, medical colleges, and hospitals exhibited distinct cluster combinations. But variations were also seen for the sites based on the samples collected in two different seasons. The spatial distribution analysis revealed intriguing associations between cluster combinations and geographic positioning.

In our analysis, we’ve observed that certain locations are associated with multiple clusters, such as 0/3, 1/3, 0/2, 1/2, 2/3, 0/1/2, and 0/2/3. After minute examination, we’ve identified that the combination of clusters 0 and 3 appears most frequently, occurring in 8 different locations, making it the most prevalent two-cluster combination. Additionally, the combination of clusters 0, 1, and 2 occurs 4 times, which is the most common three-cluster combination in our observations. The locations with the 0/3 cluster combination, we found that out of 8 different geographic locations, 6 (75%) are situated near flowing water sources such as rivers and streams, while the remaining 2 (25%) are near stagnant water sources. Similarly, for the geographic locations associated with the 0/1/2 cluster combination, we found that all four (100%) of these locations are in close proximity to medical colleges and hospitals. Different areas may yield varied viral loads due to population density and access to sanitation, potentially skewing viral presence estimates if not uniformly distributed. Viral load in wastewater can fluctuate seasonally or with rainfall, potentially affecting concentration consistency and detection sensitivity. Temperature, wastewater treatment processes, and chemical pollutants impact viral RNA stability, potentially leading to data inaccuracies in viral load measurement (Medema et al., 2020; Albastaki et al., 2021).

This study’s findings align with existing literature emphasizing the role of environmental conditions in modulating viral persistence and transmission dynamics (Smyth et al., 2022; Rimoldi et al., 2020; Wurtzer et al., 2020; Kitajima et al., 2020; Gogoi et al., 2024). Understanding these associations aids in devising targeted intervention strategies, especially in locations with higher viral prevalence, and underscores the importance of considering environmental factors in public health management strategies. Further research is warranted to explore the complex relationships between environmental conditions and viral prevalence.

## 5 Conclusion

In summary, this study leveraged unsupervised machine learning algorithms to analyze physicochemical parameters, heavy metal content, and SARS-CoV-2 positivity data from wastewater samples collected from 28 sites in Jorhat district, Assam, India. This innovative approach marked the first exploration of the influence of environmental variables on SARS-CoV-2 positivity in the North Eastern region of India. The K-means clustering algorithm effectively partitioned the data into four clusters, revealing significant associations between environmental conditions and viral prevalence. Notably, Cluster 2 exhibited the highest SARS-CoV-2 positivity rates, particularly during the rainy season, and was characterized by higher temperatures, lower turbidity, and increased levels of iron and chloride. Conversely, Cluster 0, with higher turbidity and certain mineral concentrations, showed the lowest positivity rates, especially during the dry season.

The spatial distribution analysis underscored the potential impact of geographic and seasonal variations on viral prevalence, with sample sites near flowing water sources and medical institutions consistently aligning with specific cluster combinations. These findings align with existing literature on the role of environmental conditions in viral persistence and transmission dynamics.

In conclusion, our findings highlight the critical role of environmental factors in shaping SARS-CoV-2 prevalence patterns in wastewater. This study underscores the importance of integrating environmental considerations into public health surveillance and intervention strategies. By demonstrating the utility of machine learning frameworks in epidemiological studies, this research provides valuable insights for targeted public health interventions in areas with higher viral prevalence. Further research is warranted to explore these relationships in different geographic and environmental contexts.

## Data Availability

The datasets presented in this study can be found in online repositories. The names of the repository/repositories and accession number(s) can be found in the article/Supplementary Material.
