# Supplementary material for: Leveraging environmental microbial indicators in wastewater for data-driven disease diagnostics
Source: Front Bioeng Biotechnol. 2024 Nov 25;12:1508964. doi: 10.3389/fbioe.2024.1508964 (PMC11625583; doi:10.3389/fbioe.2024.1508964)
Supplement: Supplementary file 1 [file DataSheet1.docx]

**SUPPLEMENTARY MATERIAL**

**Leveraging Environmental Microbial Indicators in Wastewater for Data-Driven Disease Diagnostics**

Gayatri Gogoi[a](#A)[d](#bookmark), Sarangthem Dinamani Singh[a](#A)[d](#c), Devpratim Koch^[a](#a)^[^d^](#c), Emon Kalyan^[a](#a)^, Rashmi Rani Boro[a](#A), Aradhana Devi^c^, Hridoy Jyoti Mahanta[b](#b)d[*](#star), Pankaj Bharali[a](#A)^d^[*](#star)

**Author Affiliations:**

^a^Centre for Infectious Diseases, Biological Sciences and Technology Division, CSIR-North East Institute of Science and Technology, Jorhat, Assam, India-785006

^b^Advanced Computation and Data Sciences Division, CSIR-North East Institute of Science and Technology, Jorhat, Assam, India-785006

^c^Materials Sciences and Technology Division, CSIR-North East Institute of Science and Technology, Jorhat, Assam, India-785006

^d^Academy of Scientific and Innovative Research (AcSIR), Ghaziabad-201002, India

**^*^Corresponding Authors:**

Dr Pankaj Bharali, Centre for Infectious Diseases, Biological Sciences and Technology Division, CSIR-North East Institute of Science and Technology (CSIR-NEIST), Jorhat, Assam, India-785006 ([pankajbharali98@gmail.com](mailto:pankajbharali98@gmail.com)/ [pbharali@neist.res.in](mailto:pbharali@neist.res.in)).

Dr Hridoy Jyoti Mahanta, Advanced Computation and Data Sciences Division, CSIR-North East Institute of Science and Technology (CSIR-NEIST), Jorhat, Assam, India-785006 ([hridoy69@gmail.com](mailto:hridoy69@gmail.com)/ [hridoy@neist.res.in](mailto:hridoy@neist.res.in)).

**Supplementary Table S1** Sample collection sites and season wise no. of collected samples

**Supplementary Table S2** Cluster distributions in different locations

**Supplementary Figure S1**: Sequential processing steps for wastewater sample preparation: 1. Pasteurization - heat treatment to neutralize microbes. 2. Sample filtration - removal of particulate matter for clarity. 3. Precipitation using PEG-NaCl method - selective precipitation of target compounds for further analysis.

**Supplementary Figure S2.** (A) In the current study, the red-coloured spot on the map of India represents the sample collection site i.e., Jorhat, Assam. (B) The green colour on the map specifically denotes Assam, while the blue areas correspond to other states within the North East India. (C) Within the Jorhat District of Assam, a total of 28 different sample collection sites are indicated by the red spots on the map, which is a visual representation of Assam on the larger India map.

**Supplementary Figure S3.** The alluvial plot provides a visual representation of the distribution of different SARS-CoV-2 genes i.e. ORF 1ab, N, and RNAseP, along with the corresponding results (positive and negative) observed in wastewater samples. The red blocks depict the Cycle Threshold (Ct) values for each gene in the samples, while the green blocks indicate the absence of a Ct value (ND - No Detection) in the three genes.

**Supplementary Figure S4**: The elbow method in K-means clustering elegantly reveals the ideal cluster count for our dataset. As clusters increase, variance consistently decreases, yet beyond the point of four clusters, the rate of reduction becomes gradual, forming an unmistakable 'elbow' in the curve. This distinct bend signifies that 4 clusters adeptly encapsulate the dataset's inherent patterns, making it the optimal choice for our current study.

**Supplementary Figure S5**: Cluster wise variation analysis of physicochemical parameters during wastewater surveillance in the current study. Blue colour depicted Standard Deviation and Green colour depicted Mean of the dataset.

**Supplementary Figure S6**: Cluster wise variation analysis of Mineral contents of wastewater during wastewater surveillance in the current study. Red colour depicted Standard Deviation and Green colour depicted Mean of the dataset.

**Supplementary Figure S7**: Cluster wise variation analysis of Heavy meatal content of wastewater during wastewater surveillance in the current study. Yellow colour depicted Standard Deviation and blue colour depicted Mean of the dataset.

**Table S1** Sample collection sites and season wise no. of collected samples

| **Location Name** | **Sites** | **Total Sample** | **Rainy season** | **Dry season** |
| --- | --- | --- | --- | --- |
| Nagabat veterinary (Border area) | H S | 16 | 8 | 8 |
| Nagabat-Sukanjan Road (Drain) | R S | 16 | 8 | 8 |
| Satyam Hospital & Research Center | H S | 16 | 8 | 8 |
| Titabor Sub-Divisional Civil Hospital | H S | 16 | 8 | 8 |
| Kuhum stream | Rv S | 16 | 8 | 8 |
| Mariani Railway Station | R S | 16 | 8 | 8 |
| Gatoonga Tea estate | R S | 16 | 8 | 8 |
| Mariani Residential Colony | R S | 16 | 8 | 8 |
| Kamarbandha road | R S | 16 | 8 | 8 |
| Kakodonga | Rv S | 16 | 8 | 8 |
| CID, CSIR-NEIST | R S | 16 | 8 | 8 |
| CSIR NEIST,Girl's Hostel Drain | R S | 16 | 8 | 8 |
| CSIR-NEIST, Canteen Drain | R S | 16 | 8 | 8 |
| NEEP PARK, CSIR-NEIST | R S | 16 | 8 | 8 |
| JMCH, Resident outlet | H S | 16 | 8 | 8 |
| JMCH, Hospital Outlet | H S | 16 | 8 | 8 |
| Tarajan, Kakoty gaon | H S | 16 | 8 | 8 |
| Athuvoga Bridge, Bhetamora | H S | 16 | 8 | 8 |
| Gorumora Hospital | H S | 16 | 8 | 8 |
| Bhogdoi river | Rv S | 16 | 8 | 8 |
| Khangia Gaon,Malow Ali Road | R S | 16 | 8 | 8 |
| Debanath Sharma mini PHC | H S | 16 | 8 | 8 |
| Elengmora | R S | 16 | 8 | 8 |
| Teok Tea Estate, Hospital | H S | 16 | 8 | 8 |
| FRU, Teok | R S | 16 | 8 | 8 |
| Baghmora | Rv S | 16 | 8 | 8 |
| Jhanji, Jorhat-Sibsagar border | Rv S | 16 | 8 | 8 |
| Nimatighat | Rv S | 16 | 8 | 8 |

*H S; Hospital Site, R S; Residential Site, Rv S; River Site

**Table S2** Cluster distributions in different locations

| **Location Name** | **Cluster 0** | **Cluster 1** | **cluster 2** | **Cluster 3** |
| --- | --- | --- | --- | --- |
| Nagabat veterinary (Border area) |  | P |  | P |
| Nagabat-Sukanjan Road (Drain) |  |  | P |  |
| Satyam Hospital & Research Center |  | P |  |  |
| Titabor Sub-Divisional Civil Hospital | P |  | P |  |
| Kuhum tea estate hospital drainage | P |  |  | P |
| Mariani Railway Station | P |  | P |  |
| Gatoonga Tea estate |  |  | P | P |
| Mariani Residential Colony | P |  | P |  |
| Kamarbandha road | P |  |  | P |
| CID, CSIR-NEIST | P |  |  | P |
| CSIR NEIST,Girl's Hostel Drain |  |  |  | P |
| CSIR-NEIST, Canteen Drain | P |  |  |  |
| NEEP PARK, CSIR-NEIST |  |  |  | P |
| JMCH, Resident outlet | P |  | P |  |
| JMCH, Hospital Outlet | P | P | P |  |
| Tarajan, Kakoty gaon | P | P | P |  |
| Athuvoga Bridge, Bhetamora | P | P | P |  |
| Gorumora Hospital |  | P | P |  |
| Bhogdoi river | P |  |  | P |
| Khangia Gaon,Malow Ali Road |  |  |  | P |
| Debanath Sharma mini PHC |  | P |  | P |
| Elengmora | P |  |  |  |
| Teok Tea Estate, Hospital | P | P | P |  |
| FRU, Teok | P |  |  | P |
| Baghmora | P |  |  | P |
| Jhanji, Jorhat-Sibsagar border | P |  |  | P |
| Nimatighat | P |  |  | P |

*P: Present; Blank: Absent

*P: Present; Blank: Absent


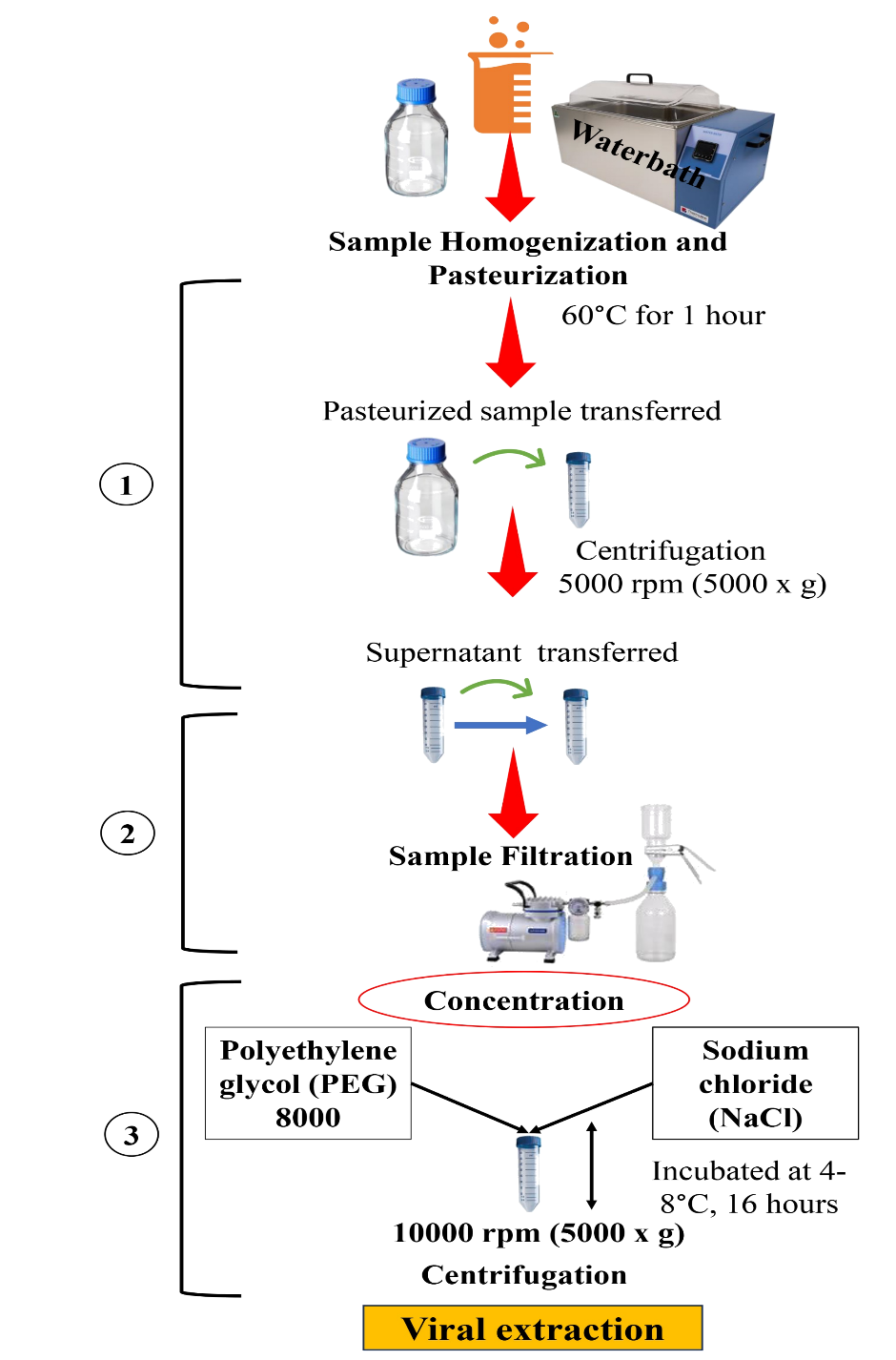


**Figure S1.** Sequential processing steps for wastewater sample preparation: 1. Pasteurization - heat treatment to neutralize microbes. 2. Sample filtration - removal of particulate matter for clarity. 3. Precipitation using PEG-NaCl method - selective precipitation of target compounds for further analysis.


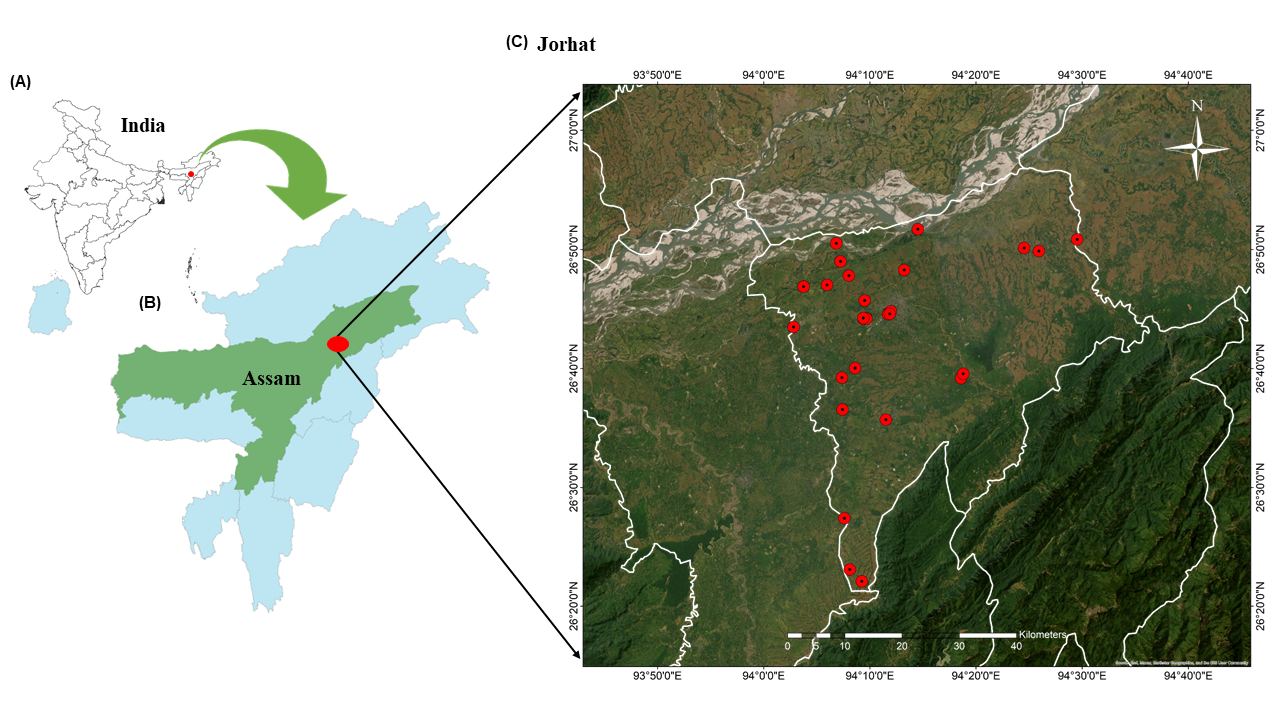


**Figure S2**. (A) In the current study, the red-coloured spot on the map of India represents the sample collection site i.e., Jorhat, Assam. (B) The green colour on the map specifically denotes Assam, while the blue areas correspond to other states within the North East India. (C) Within the Jorhat District of Assam, a total of 28 different sample collection sites are indicated by the red spots on the map, which is a visual representation of Assam on the larger India map.


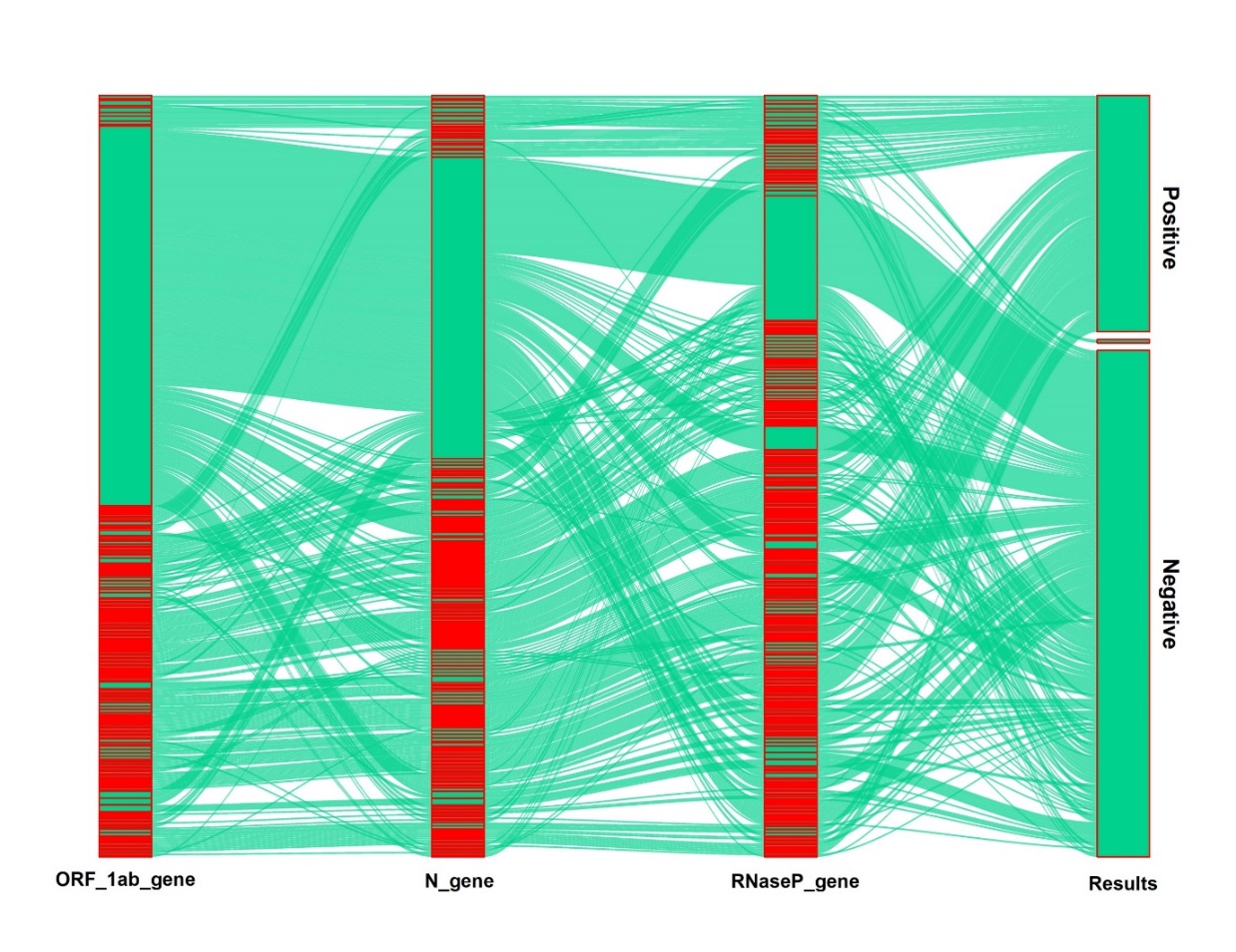


**Figure S3.** The alluvial plot provides a visual representation of the distribution of different SARS-CoV-2 genes i.e. ORF 1ab, N, and RNAseP, along with the corresponding results (positive and negative) observed in wastewater samples. The red blocks depict the Cycle Threshold (Ct) values for each gene in the samples, while the green blocks indicate the absence of a Ct value (ND - No Detection) in the three genes.


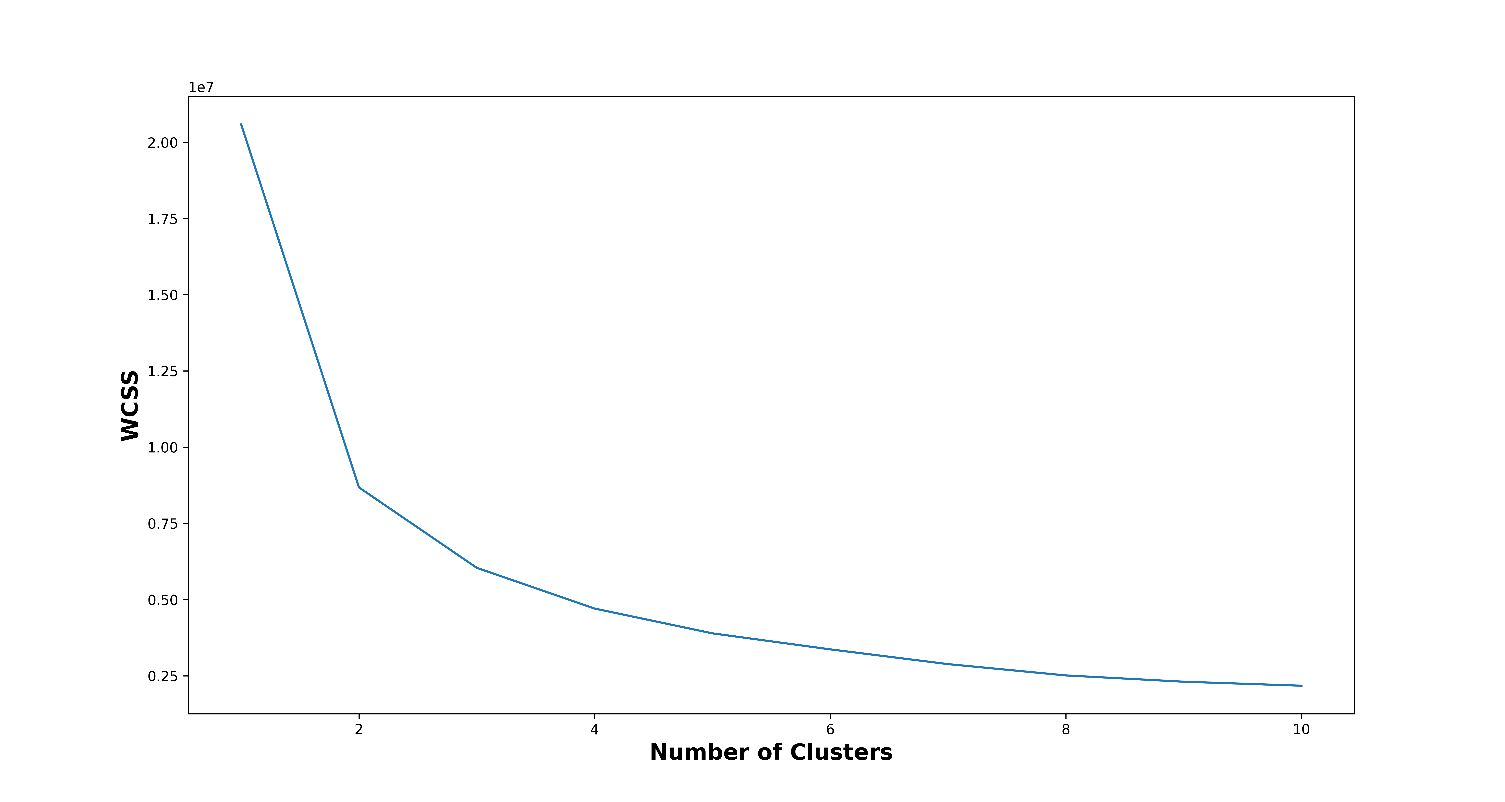


Figure S4: The elbow method in K-means clustering elegantly reveals the ideal cluster count for our dataset. As clusters increase, variance consistently decreases, yet beyond the point of four clusters, the rate of reduction becomes gradual, forming an unmistakable 'elbow' in the curve. This distinct bend signifies that 4 clusters adeptly encapsulate the dataset's inherent patterns, making it the optimal choice for our current study.


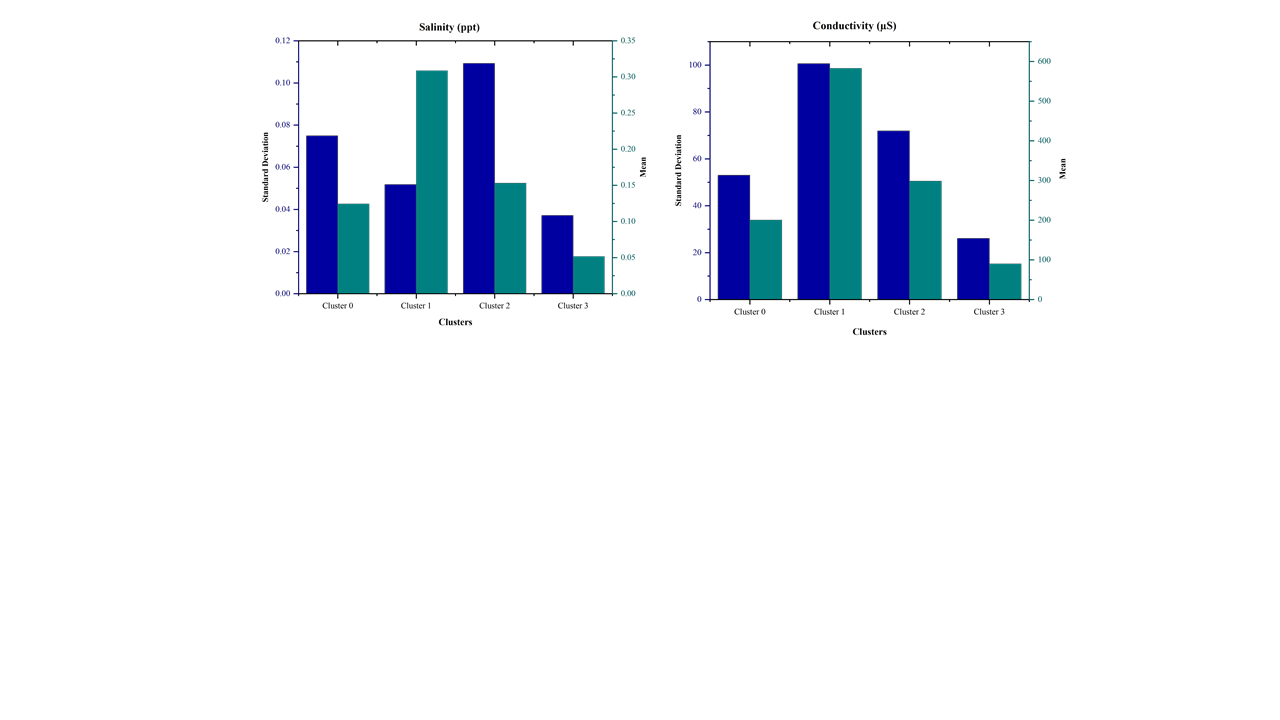

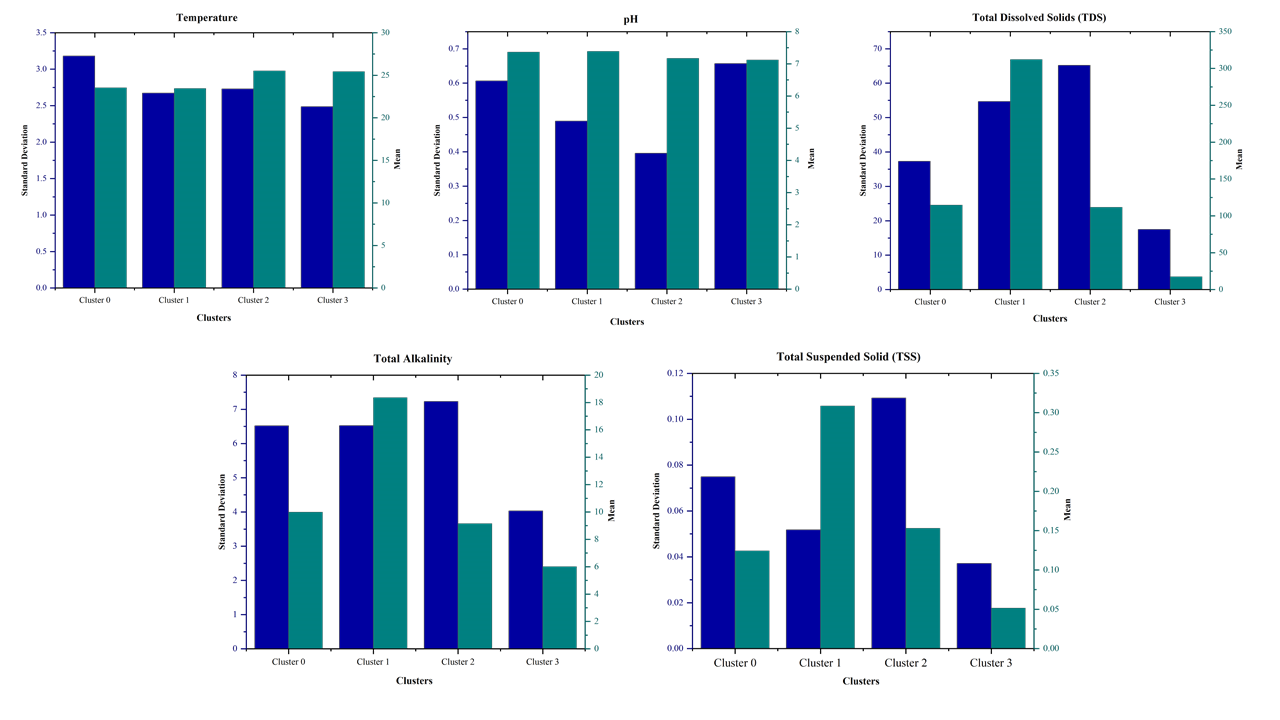


Figure S5: Cluster wise variation analysis of physicochemical parameters during wastewater surveillance in the current study. Blue colour depicted Standard Deviation and Green colour depicted Mean of the dataset


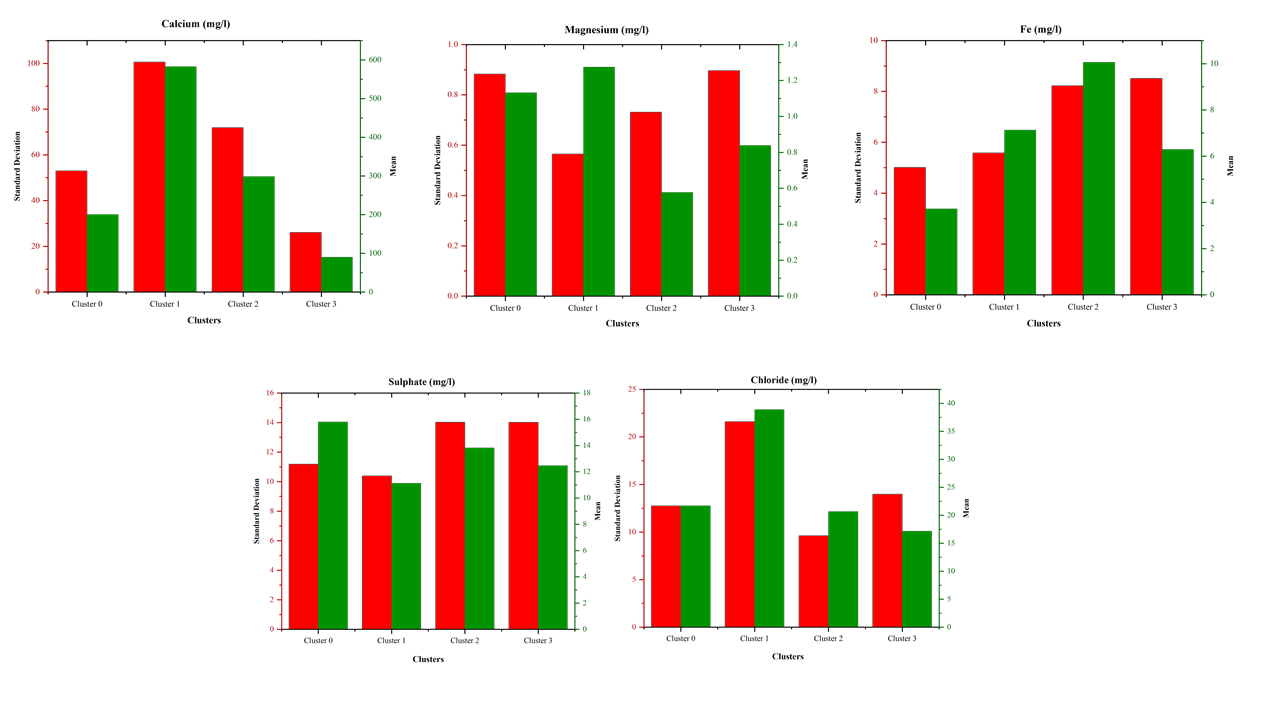


Figure S6: Cluster wise variation analysis of Mineral contents of wastewater during wastewater surveillance in the current study. Red colour depicted Standard Deviation and Green colour depicted Mean of the dataset


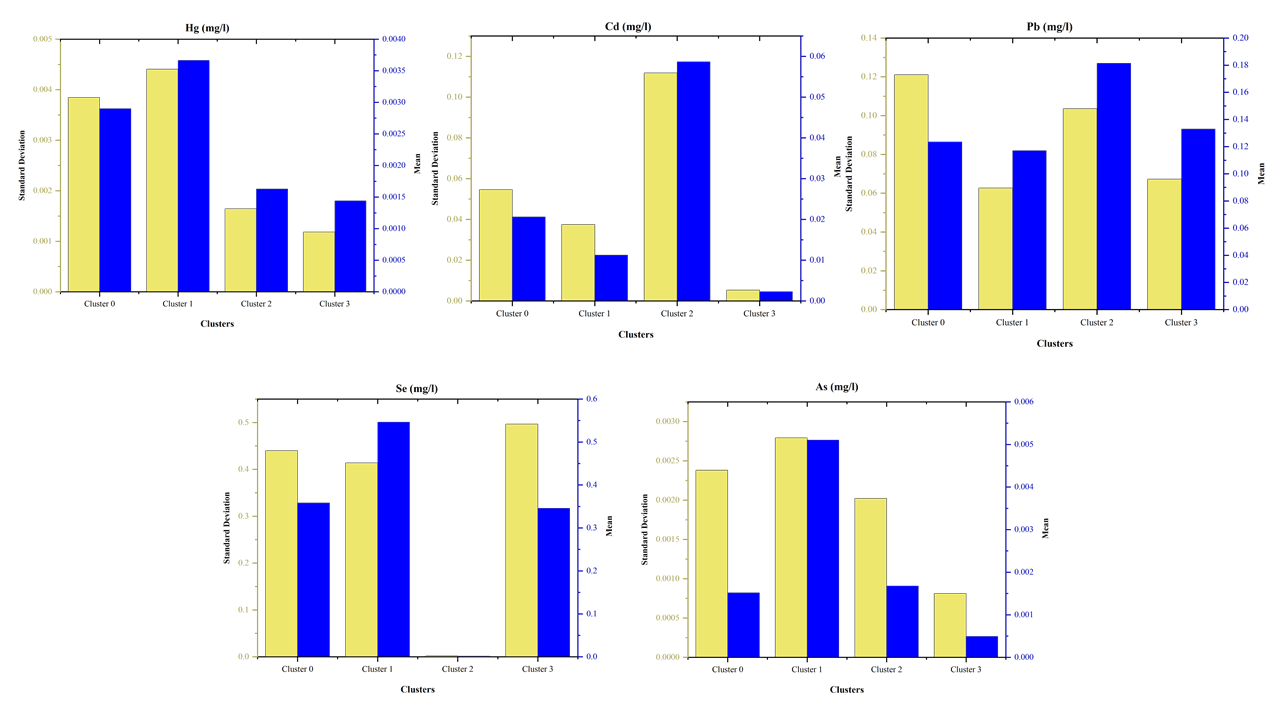
Figure S7: Cluster wise variation analysis of Heavy meatal content of wastewater during wastewater surveillance in the current study. Yellow colour depicted Standard Deviation and blue colour depicted Mean of the dataset.
